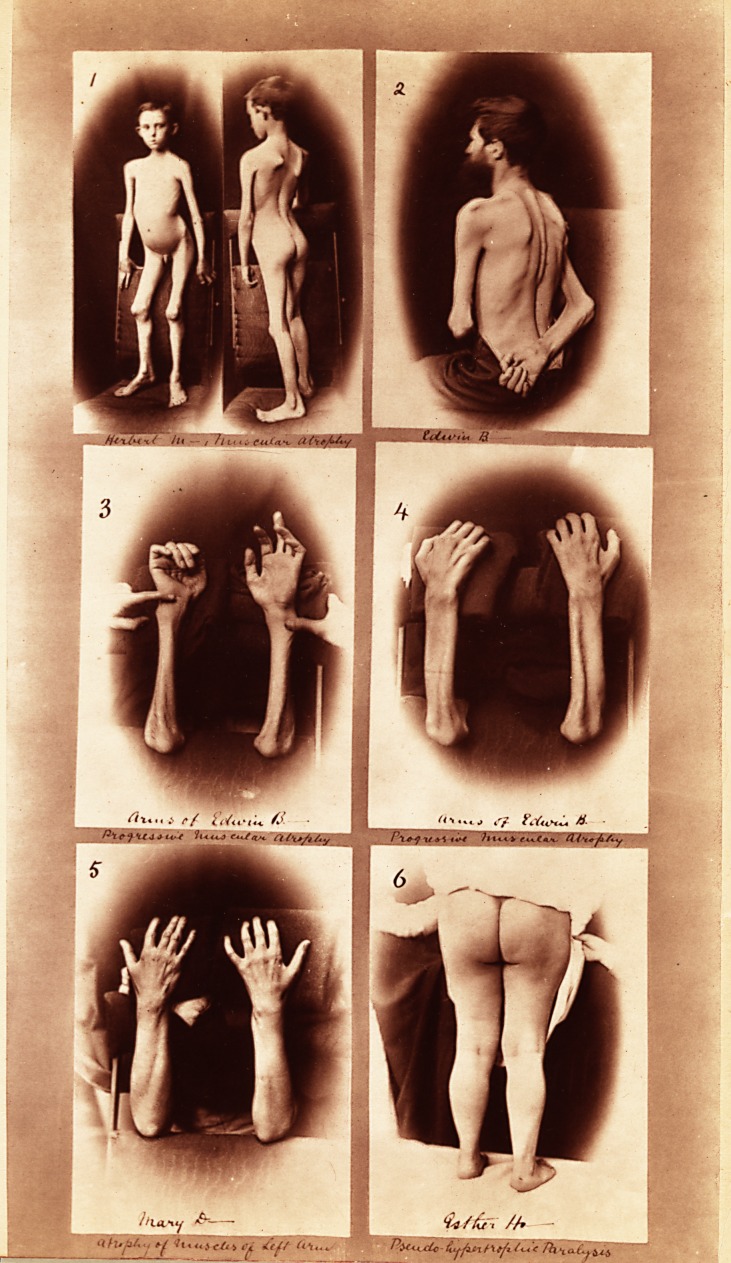# Cases of Muscular Atropy and Degeneration

**Published:** 1883-12

**Authors:** J. Fenton Evans

**Affiliations:** House Physician, Bristol Royal Infirmary


### Clinical Records.

---

### CASES OF MUSCULAR ATROPHY AND DEGENERATION. By J. Fenton Evans, M.B., House Physician, Bristol Royal Infirmary.

The accompanying photographs are of some interesting cases of atrophy and degeneration of muscles which have been in the medical wards of the Bristol Royal Infirmary during the past six months :—

No. 1.—Herbert M. Progressive atrophy of most of the muscles of the trunk, chiefly the abdominal and scapular muscles.

No. 2.—Edwin B. Progressive muscular atrophy, showing the wasting of the erector spinæ and consequent prominence of the spines of the vertebræ.

No. 3.—Edwin B. Fore-arms and hands showing wasting of the flexor and extensor groups of muscles on either side of the elbow, and of the thenar and hypothenar eminences of the palm. As is shown in the photograph, the fingers of the left hand can be straightened to a varying extent, but not those of the right hand, which presents accordingly the "main en griffe."

No. 4.—Edwin B. Fore-arms showing a deep groove between the radius and ulna, due to atrophy of the extensor muscles, also grooves between the metacarpal bones caused by wasting of the dorsal interossei.

No. 5.—Mary D. Both arms, the atrophy limited to muscles of left fore-arm and hand.

No. 6.—Esther H. Pseudo-hypertrophic paralysis, excessive development of lower limbs.

Herbert M., æt. 10 years, under Dr. Shingleton Smith, is said to have always been weakly since a fall at $2\frac{1}{2}$ years of age, his condition getting steadily worse during the last four years, unable to go to school during the last six months. On attempting to walk he frequently falls, bruising himself; can use his arms well. The face entirely lacks expression, the lower jaw and lip hanging so that the mouth is constantly open. The eyelids cannot be completely closed, a chink a quarter of an inch wide being left after the strongest effort. The tongue is protruded evenly. There is no difficulty in mastication, deglutition, defæcation or micturition.

As he stands the abdomen bulges in a remarkable manner, the spine being drawn back and the thorax raised. This is due to the atrophy of the abdominal muscles and unopposed action of the erector spinæ, the viscera protruding owing to the laxity of the abdominal wall. Very little action of the abdominal muscles occurs on stooping.

The muscles connecting the scapulæ to the trunk appear to have suffered most, the rhomboidei and trapezii being almost absent, thereby allowing the scapula to be tilted up and out in a curious wing-like manner from the shoulder. The muscles proper of the scapula are not much affected.

The muscles of the limbs are wasted, more in the upper than the lower extremity, more in the thighs than below the knee; those of the hands and feet escaping the process of atrophy almost entirely, the case presenting in this a marked difference to ordinary progressive muscular atrophy, as also in the absence of fibrillary twitchings in any of the muscles.

The pectoral muscles are almost absent and do not respond to the Faradic current, by which contractions can be produced to a varying extent in all the other muscles.

Patellar reflex and ankle-clonus both absent. The organs are all normal. Retinæ and discs normal.

Patient was in the house three weeks, during which time he gained strength and 4 lbs. in weight, and is now improving as an out-patient.

The etiology of this case presents some difficulty. There was no want of intelligence, no affection of a cranial nerve, and no error of sensibility. The want of power proceeded *pari passu* with the muscular atrophy, there had never been any febrile symptom or acute attack, so that consequently the case is most akin to progressive muscular atrophy, although unlike it in the points already mentioned.

2. Edwin B., æt. 42 years, cabinet-maker, a patient under Dr. Shingleton Smith, has suffered for the last four years with increasing weakness and muscular atrophy. At first his attention was excited by the fact that he frequently dropped his tools after taking them up, and shortly afterwards was unable to use the "scraper." The fingers soon became flexed and the wrist dropped, together with a wasting of the balls of the thumbs. The wasting of the muscles has increased up to the present time, those of the upper limbs, spine, and face having suffered chiefly. In the hands the thenar and hypothenar eminences are wanting, the dorsal interossei and the muscles of the fore-arm are replaced by deep grooves. On either side of the spine is a deep groove, as also beneath the clavicles, due to atrophy of muscles. Fib-

Herbert M—, Muscular atrophy

Edwin B—

Arms of Edwin B—
Progressive Muscular atrophy

Arms of Edwin B—
Progressive Muscular atrophy

Mary D—

atrophy of Muscles of Left Arm

Esther H—
Pseudo-hypertrophic Paralysis rillary twitchings are constantly going on. Most of the muscles respond to the Faradic current.

The pupils are pin-point, not acting with accommodation, and nearly insensitive to light. Optic discs: left, slightly indistinct; right, no outline; veins of each retina distended. Posterior staphyloma at upper part of right disc.

Sexual inclination has been absent during last year. Micturition is frequently difficult from want of power in bladder.

The cutaneous reflexes are increased, especially the right cremasteric, abdominal and plantar. No ankle-clonus. Patellar reflex normal.

Patient constantly suffers from crampy pains, almost like those of posterior sclerosis. Nine years ago he had rheumatic fever, when most of his joints were affected.

This case, one of advanced progressive muscular atrophy, is interesting from the presence of the oculo-pupillary phenomena, indicating some affection of the cervical sympathetic in addition to that of the anterior cornua. It affords also a contrast to the case of Sarah M., æt. 40, who was admitted under Dr. Waldo with partial paralysis of the four limbs of one week's duration. This gradually increased for a time after admission, being slowly followed in from two to three months' time by restoration of power, first in the arms, and now slowly in the legs; but this return of motor power was accompanied by atrophy of the muscles, the hands presenting the characteristic "main en griffe," so marked in the case of Edwin B. But the history of the case is one of atrophy occurring subsequent to the paralysis, and with the return of voluntary muscular action instead of *pari passu* with its loss. The absence of fibrillary twitching, and the inability of the muscles in their present or atrophic condition to respond to the Faradic current, leave no doubt that the case of Sarah M. is one of chronic atrophic spinal paralysis.

5. Mary D., æt. 36, domestic servant, a patient under Dr. Shaw. The atrophy in this case is strictly limited to the left fore-arm and hand. Both the flexor and extensor group is atrophied, but chiefly the flexor. In the hand the thenar and hypothenar eminences and dorsal interossei are much wasted, the fingers slightly flexed upon the palm, the heads of the metacarpal bones prominent anteriorly, the hand presenting the appearance of "main en griffe." Fibrillary twitchings of the affected muscles are constantly occurring, and specially in the first dorsal interosseous. The left hand is $\frac{4}{5}^{\circ}$ colder than the right, the left axilla 1° colder than the right. There is considerable impairment of thermal and tactile sense. The wasted muscles all respond to a Faradic current, but to a less extent than do those of the right fore-arm to a current of equal strength.

This condition has only prevented the patient from working during the last three months, the left hand and fore-arm often giving way of late; but she has been sensible of a growing weakness in the left arm for at least ten years. The wasting of the hand was pointed out to her about three years ago by a friend. No history of injury to the brachial or other nerves, or evidence of neuroma. General health excellent.

6.—Esther H., æt. 17, and Mary H., æt. 16, sisters; patients under Dr. Shaw; both suffering from pseudo-hypertrophic paralysis, the paralysis and hypertrophy affecting the lower extremities chiefly.

Both sisters enjoyed good health until four years ago, when both had scarlet fever, from which they recovered. Six months subsequent to this, Esther, the elder, began to fail in strength, and noticed that the calves of her legs were becoming larger and hard; three months later Mary H. also became affected in the same way. Family history good, healthy brothers and sisters. Mary H. is wanting in intelligence.

Both sisters are very short, 4 feet 8 inches in height, of strumous appearance, puffy face, teeth pegged and separated. Esther H. is unable to walk or even stand by herself; Mary H. is able to stand and walk a little with the characteristic waddling gait and some amount of talipes equinus, but not able to raise herself from the kneeling posture. As can be seen from the photograph both the buttocks and calves are much hypertrophied, the latter measuring thirteen inches in circumference in each case. Some paralysis and hypertrophy of the deltoids, so that neither patient is able to raise her arm to a level with the shoulder. The affected muscles respond feebly to the interrupted current. Patellar reflex wanting. No ankle-clonus.

An hereditary predisposition to this disease has been frequently observed, although not in direct descent as a rule, since death generally occurs in youth. Children of the same family are sometimes affected, as in the cases given. The rarity of the disease in the United States is interesting; Hammond, in 1876, had only seen two cases, and mentions that only seven others had been reported up to that time.

---

I am indebted to Dr. Shingleton Smith and Dr. Shaw for the accompanying photograph and for permission to publish these cases.

## Figures and Tables

**1 2 3 4 5 6 f1:**